# A sound methodology: Measuring experiences of violent conflict through audio self-interviews

**DOI:** 10.1016/j.econlet.2024.111879

**Published:** 2024-09

**Authors:** Sophie von Russdorf, Laura Ahlborn, Alessandra Hidalgo-Arestegui, Gerald McQuade, Marta Favara

**Affiliations:** a Oxford Department of International Development, University of Oxford, Oxford, UK; b Paris School of Economics, Université Paris 1 Panthéon Sorbonne, Paris, France; c Lancaster University Management School, Lancaster University, Lancaster, UK

**Keywords:** Conflict, Measurement, Survey methods, Tigray, Ethiopia

## Abstract

This paper investigates the impact of different survey administration methods on the disclosure of sensitive or traumatic experiences. Respondents of a pilot study in Ethiopia were randomly assigned to answer questions either using audio computer-assisted self-interviewing (ACASI) or as part of a face-to-face (FtF) enumerator-based interview. Results indicate that ACASI led to higher disclosure rates of conflict-related experiences, particularly for the most sensitive questions, i.e., when either the respondent or a close friend or family member was the victim, or when the trauma suffered was more severe. ACASI offers a viable solution to measure traumatic conflict-related experience exposure in low-literacy settings, overcoming the underestimation problem commonly observed when using standard survey methods.

## Highlights

• Face-to-face (FtF) disclosure of sensitive experiences may suffer from reporting bias.
• Audio computer-assisted self-interviewing (ACASI) may be a viable alternative.
• We test FfF vs. ACASI on eliciting information about conflict exposure in Ethiopia.
• ACASI increases reporting of experiences at both intensive and extensive margins.
• Reporting under ACASI is more likely when the experience is more severe.

## 1 Introduction

Despite advances in survey methodology, obtaining accurate data on the experiences of individuals exposed to conflicts remains challenging. When asking sensitive questions, or where there is a risk of adverse consequences if answers were made public (Brück et al., 2013), respondents may refuse to disclose or systematically under- or over-report certain socially (un-)desirable behaviours or experiences (Tourangeau and Yan, 2007), obfuscating the true impacts of conflict.

This paper presents findings from a randomized experiment in Ethiopia, afflicted since November 2020 by atrocious conflict centred in the Tigray region between the federal government and various regional forces, including the Tigray People’s Liberation Front. We aim to assess, for the first time, the effectiveness of audio computer-assisted self-interviewing (ACASI), where respondents use headphones to listen to pre-recorded questions and record responses on a tablet, compared to face-to-face (FtF) enumerator-based interviewing in eliciting information on individual exposure to violent conflict. ACASI is intended to mitigate the risk of under-reporting by increasing privacy and confidentiality. Furthermore, compared to other self-interview (SI) methods, respondents are not required to read or write answers, potentially reducing errors in reporting and misinterpreting, especially in low-literacy contexts (Gnambs and Kaspar, 2015).

Overall, our results suggest participants in the ACASI treatment group report a greater number of experiences than those in the FtF control group, particularly when the experience is more severe and affects them or a friend/relative directly. These findings are aligned with existing evidence showing increased disclosure of sensitive behaviours, such as: drug use (Simoes et al., 2006); sexual behaviour (Phoo et al., 2022); STD prevalence (Ghanem et al., 2005) and gender-based violence (see Peterman et al., 2023 for a recent review), when comparing ACASI against FtF and other SI methods.

The rest of this paper is structured as follows. Section 2 provides an overview of the data and the experimental design. Section 3 outlines the estimation strategy used. Section 4 reports our results, and Section 5 concludes.

## 2 Experimental design and measurements

This study was conducted in May 2023, during a pilot survey for the seventh round of Young Lives (YL), a longitudinal cohort study following approximately 3,000 young people and their families in Ethiopia since 2002 (Favara et al., 2022). 139 respondents were recruited across ten sites in the Tigray, Amhara, Oromia, and Addis Ababa regions, resembling the characteristics of the YL sample, a study that although not nationally representative, reflects Ethiopia’s regional and urban/rural diversity (Sanchez and Outes-Leon, 2008). The characteristics of the sample are reported in Table 1.

All respondents took part in a standard FtF interview and agreed to a self-completed questionnaire (SCQ), administered using ACASI, regarding sensitive questions on sexual and risky behaviours, and IPV. Both FtF interviews and ACASI were conducted in one of the three local languages (Amarigna, Tigrigna, Oromiffa). ACASI was administered on tablets with participants using headphones to privately listen to questions featuring gender-matched speakers. Response options were visually presented using simple shapes and colours to maintain answer confidentiality and reduce issues related to low literacy (see Appendix Figure A1).

**Table 1:** Descriptive statistics.

	Total	Control	Treated	Diff.
Age (in years)	25.33	25.41	25.24	0.17
	(3.28)	(3.27)	(3.33)	
Male	0.52	0.54	0.49	0.05
	(0.50)	(0.50)	(0.50)	
Completed secondary education	0.40	0.45	0.33	0.12
	(0.49)	(0.50)	(0.47)	
Employed in last year	0.80	0.82	0.76	0.06
	(0.40)	(0.38)	(0.43)	
Wealth index score	0.44	0.45	0.42	0.03
	(0.16)	(0.17)	(0.14)	
Married	0.29	0.31	0.27	0.04
	(0.46)	(0.47)	(0.45)	
Gender match between respondent and fieldworker	0.56	0.55	0.56	−0.01
	(0.50)	(0.50)	(0.50)	
Rural	0.41	0.35	0.49	−0.14
	(0.49)	(0.48)	(0.50)	
**Region**				
Addis	0.14	0.15	0.13	0.02
	(0.35)	(0.36)	(0.34)	
Amhara	0.28	0.24	0.33	−0.08
	(0.45)	(0.43)	(0.47)	
Oromiya	0.27	0.32	0.20	0.12
	(0.45)	(0.47)	(0.40)	
Tigray	0.31	0.28	0.35	−0.06
	(0.46)	(0.45)	(0.48)	

Observations	129	74	55	129

*Notes:*${}^{*}$p< 0.1, ${}^{**}$p< 0.05, ${}^{***}$p< 0.01. Standard deviations are in parentheses.

**Table 2:** Overview of conflict experiences module.

Question	Random -ised	FtF only	ACASI only
1. Experienced a serious food shortage		X	
2. Been unable to continue with your work or find work		X	
3. Been unable to access health care		X	
4. Been unable to continue with your education or that of your children		X	
5. Been unable to access utilities (water, electricity)		X	
6. Been unable to access communication infrastructure (telephone, mobile phone, internet) or banking infrastructure (banking, mobile banking, savings)		X	
7. Been asked to work for free		X	
8. Been verbally threatened or insulted		X	
9. Been seriously injured/wounded	X		
10. One of your family members/friends has been seriously injured/wounded	X		
11. One of your family members/friends disappeared	X		
12. One of your family members/friends has died as a result of the conflict	X		
13. Experienced any kind of (attempted) physical violence, such as being attacked with a weapon, being shot at (not necessarily wounded), or chased	X		
14. Witnessed people experiencing any kind of (attempted) physical violence, such as being attacked with a weapon, being shot at (not necessarily wounded), or chased	X		
15. Witnessed people being killed	X		
16. Witnessed people experiencing any kind of (attempted) sexual violence	X		
17. Been encouraged, asked, or forced to commit any violent act	X		
18. Been encouraged, asked, or forced to join an armed movement/rebel group/militia		X	
19. Experience any kind of (attempted) sexual violence			X

*Notes:* The conflict experiences modules in the FtF and the ACASI were both introduced by asking the respondents “about some of the experiences [they] may have had [...] since November 2020, when the conflict started.” In the FtF, if the answer to a given question was yes, it was followed by the question “Did you experience it due to conflict?”. In the ACASI, each question directly referenced the conflict, ending with the addition “...as a result of the current conflict?”. Question 18 was only asked to the respondents who answered questions 9 to 17 as part of the FtF interview.

Finally, respondents were asked about 19 conflict-related situations experienced since November 2020, as listed in Table 2.^2^ 11 items were identified as sensitive. Of these, one was only administered in FtF and one, deemed too sensitive for FtF, was administered only in ACASI.

For the remaining 9 items, respondents were randomized to complete them as part of the ACASI (N = 65, treatment group TG) or FtF interview (N = 74, control group CG). Evidence suggests that randomization was successfully implemented, given the good balance in characteristics (Table 1) and non-sensitive placebo items across samples (Table B1).^3^

**Table 3:** Effect of ACASI on reporting conflict-related experiences.

	ACASI	Control mean (S.D.)
*Panel A: Index results*		
Total reported experiences	0.958	1.72
	(0.353)***	(2.38)
Reports any experiences	0.181	0.46
	(0.073)**	(0.50)

*Panel B: Individual item results*		
**Self - Victim**		
Seriously wounded/injured	0.081	0.03
	(0.045)*	(0.16)
Experienced physical violence	0.170	0.11
	(0.065)***	(0.31)
**Family - Victim**		
Family member/friend has been seriously wounded/injured	0.051	0.31
	(0.074)	(0.47)
Family member/friend disappeared	0.138	0.28
	(0.068)**	(0.45)
Family member/friend died	0.159	0.24
	(0.073)**	(0.43)
**Anyone - Victim**		
Witnessed physical violence	0.205	0.32
	(0.075)***	(0.47)
Witnessed someone being killed	0.033	0.28
	(0.070)	(0.45)
Witnessed sexual violence	0.105	0.08
	(0.061)*	(0.27)
**Self - Perpetrator**		
Asked/forced to commit violent act	0.015	0.05
	(0.051)	(0.23)

Controls	Yes	
N	129	

*Notes:*${}^{*}$p< 0.1, ${}^{**}$p< 0.05, ${}^{***}$p< 0.01. Robust standard errors are in parentheses. All controls included.

## 3 Empirical strategy

We assess how different survey administration methods impact misreporting bias, comparing reporting patterns across FtF and ACASI. Given random assignment to survey methods, any difference in outcomes is assumed to be due to the ACASI method treatment. Following Cullen (2023), we estimate using OLS:

(1) $$Y_{i}=\alpha +\rho T_{i}+\beta^{\prime }X_{i}+{ɛ}_{i}$$

Where ρ is the average treatment effect (ATE) $T_{i}$ on (1) the number of conflict experiences reported and (2) the probability of reporting any experience. All specifications are estimated with robust standard errors using a balanced sample with no missing values in our main outcomes of interest (N = 129). We include a vector $X_{i}$ of controls including the region (Addis Ababa, Amhara, Oromia, and Tigray); a dummy for rural/urban sites; if the respondent moved after the beginning of the conflict; if they completed secondary education; age in years; and a multidimensional wealth index (Briones, 2017).

## 4 Results

In panel A of Table 3, we present the main results from estimating Eq. (1) on the two outcomes of interest.^4^ We find that, on average, participants in the ACASI TG report 0.958 conflict experiences more than those in the CG and have an 18.1 percentage point (p.p.) increase in their probability of reporting any experience.

Our results are robust when we exclude all control variables (Table B3), drop respondents who moved woredas since the start of the conflict (about 33.3%) or include fieldworker fixed effects to address potential surveyor bias (Table B4). As the distribution of the total number of experiences reported is positively skewed, with many respondents reporting zero items, we report the effect of ACASI on the inverse hyperbolic sine transformed value (Bellemare and Wichman, 2020) with no significant changes in our main results (Table B4).

In Panel B of Table 3, we provide estimates of the impact of ACASI on reporting each conflict experience individually. We observe positive effects on reporting probability across all items, although not all estimated parameters are statistically significant.^5^ Some patterns can be identified. First, the probability of reporting a traumatic experience through ACASI is higher (and more precisely estimated) compared to FtF when respondents themselves have experienced it as a victim (for example, being subject to serious injury or physical violence increasing by 8.1p.p. and 17p.p., respectively) rather than as perpetrators. Second, the more severe the trauma experienced/witnessed, the more ACASI respondents feel comfortable reporting them relative to FtF respondents. Notably, ACASI respondents are more likely to report having witnessed physical (20.5p.p.) and sexual violence (10p.p.). Third, the probability of reporting an experience through ACASI is higher if the victim is a close friend or family member. However, this effect is only significant for more severe subjects (family member/friend disappeared (13.8p.p.) or died (15.9p.p.)). Finally, we do not find strong evidence on heterogeneous treatment effects when considering the respondents’ sex, economic status and level of education.

## 5 Discussion and conclusion

We report the results of a randomized pilot experiment designed to test the impact of survey administration method (ACASI versus FtF) on eliciting information about individuals’ traumatic experiences due to violent conflict. According to our results, respondents in the ACASI treatment group are more likely to report having been a victim/witness of conflict-related traumatic experiences and tend to report a greater number of conflict experiences. Furthermore, we find that respondents are more likely to disclose if they or a close friend/family member were victims, especially if the experience was particularly severe.

Overall, our results suggest that ACASI is a viable alternative to measure exposure to armed conflict, particularly in a low-literacy setting, where traditional FtF survey methods would risk underestimating the prevalence of conflict exposure. While low computer literacy may be a concern with ACASI (Park et al., 2022), it is still feasible if properly designed. Additionally, 54.7% of our sample use the internet or social media, suggesting comfort with digital devices. Nevertheless, the use of pre-recorded questions, potentially across multiple languages and genders, might increase the time and costs of development and implementation. Finally, for ACASI to be effectively implemented at scale while preserving the quality of the data collected, one should limit the number and complexity of questions included to create a survey tool accessible to the targeted sample.

## Declaration of competing interest

None.

## Data Availability

The data that has been used is confidential.

## Appendix A Supplementary data

MMC S1 .

## Footnotes

2 Questions were sourced from the Conflict Exposure Module (Brück et al., 2013), the Adult Resilience Measure (Clark et al., 2022), and Brennan et al. (2007).

3 The differences in means for the 9 randomized questions are reported in Table B2.

4 Cumulative distributions for these outcomes are plotted separately for the CG and TG in Figure A2.

5 As a placebo test, we check for a spurious estimated effect of treatment on the non-randomized items and do not find significant differences (Table B5).
