# Supplementary material for: A sound methodology: Measuring experiences of violent conflict through audio self-interviews
Source: Econ Lett. 2024 Sep;242:None. doi: 10.1016/j.econlet.2024.111879 (PMC11639155; doi:10.1016/j.econlet.2024.111879)
Supplement: MMC S1 — . [file mmc1.pdf]

Appendices

A Additional figures

Figure A1: ACASI practice questions for participants

SAY: What country do you currently live in?

Press the blue button if your answer is China; press red button if your answer is Ethiopia. If you wish to skip the question press the sun.

- China
- Ethiopia
- I prefer not to answer

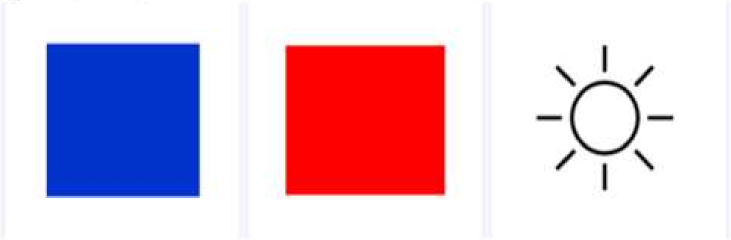

SAY: Are you under 50 years old?

Press the blue button if your answer is Yes; press red button if your answer is No. If you wish to skip the question press the sun.

- Yes
- No
- I prefer not to answer

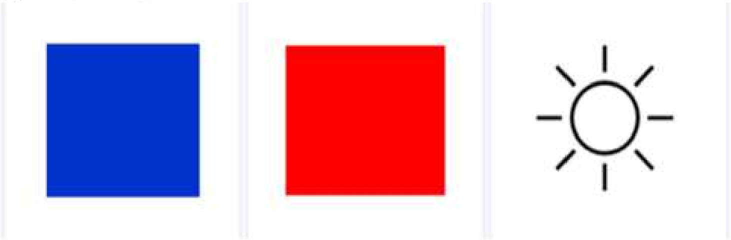

Figure A2: Cumulative distribution plots of experiences reported by survey administration method

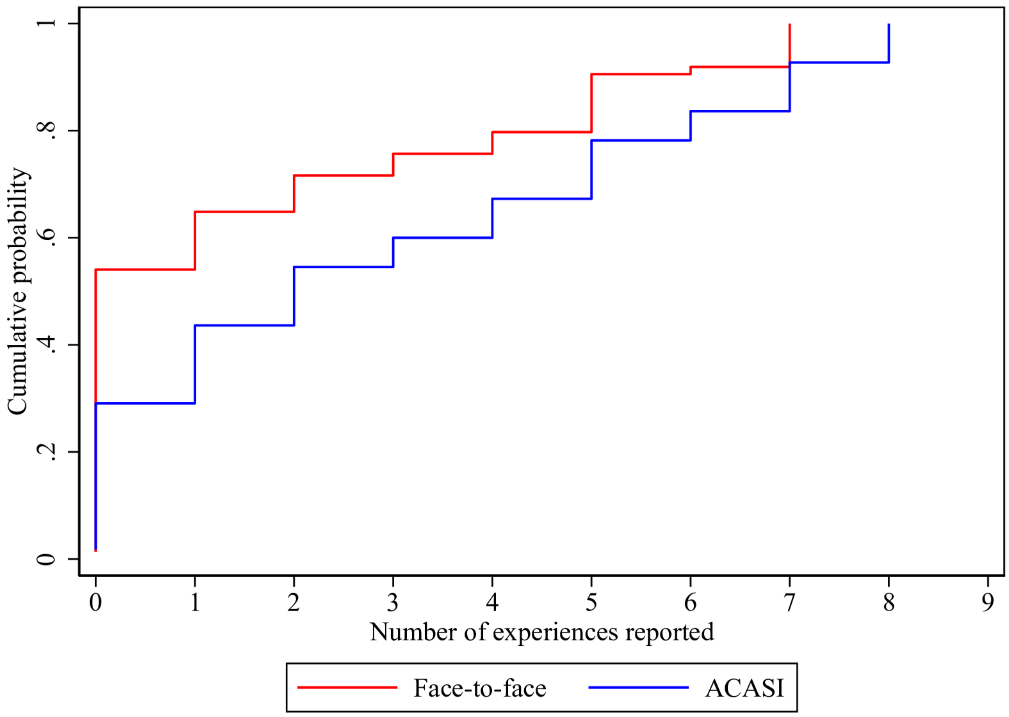

B Additional tables

Table B1: Comparison of Means - Placebo Conflict Items

	Total	Control	Treated	Diff.
Panel A: Aggregates				
Total number of (placebo) conflict experiences had	2.67	2.49	2.91	-0.42
	(2.91)	(2.93)	(2.89)	
At least one (placebo) conflict experience had	0.59	0.57	0.62	-0.05
	(0.49)	(0.50)	(0.49)	
Panel B: Individual Items				
Due to conflict: Food shortage	0.33	0.32	0.35	-0.02
	(0.47)	(0.47)	(0.48)	
Due to conflict: Unable to work	0.40	0.34	0.47	-0.13
	(0.49)	(0.48)	(0.50)	
Due to conflict: Unable to access health care	0.33	0.28	0.38	-0.10
	(0.47)	(0.45)	(0.49)	
Due to conflict: Unable to continue with education	0.28	0.31	0.24	0.07
	(0.45)	(0.47)	(0.43)	
Due to conflict: Unable to access utilities	0.42	0.36	0.49	-0.13
	(0.50)	(0.48)	(0.50)	
Due to conflict: Unable to access communications infrastructure	0.48	0.45	0.53	-0.08
	(0.50)	(0.50)	(0.50)	
Due to conflict: Asked to work for free	0.16	0.16	0.15	0.02
	(0.36)	(0.37)	(0.36)	
Due to conflict: Verbally threatened or insulted	0.22	0.22	0.22	-0.00
	(0.41)	(0.41)	(0.42)	
Due to conflict: Experienced sexual violence (ACASI only)	0.07	0.04	0.09	-0.05
	(0.25)	(0.21)	(0.29)	
Observations	129	74	55	129

Notes: * p<0.1, ** p<0.05, *** p<0.01. Standard deviations are in parentheses.

Table B2: Comparison of Means - Randomized Conflict Items

	Total	Control	Treated	Diff.
Panel A: Aggregates				
Total number of (randomised) conflict experiences had	2.22	1.72	2.91	-1.19***
	(2.62)	(2.38)	(2.78)	
At least one (randomised) conflict experience had	0.57	0.46	0.71	-0.25***
	(0.50)	(0.50)	(0.46)	
Panel B: Individual Items				
**Self - Victim**				
Seriously wounded/injured	0.06	0.03	0.11	-0.08*
	(0.24)	(0.16)	(0.31)	
Experienced physical violence	0.19	0.11	0.29	-0.18***
	(0.39)	(0.31)	(0.46)	
**Family - Victim**				
Family member/friend has been seriously wounded/injured	0.35	0.31	0.40	-0.09
	(0.48)	(0.47)	(0.49)	
Family member/friend disappeared	0.36	0.28	0.47	-0.19**
	(0.48)	(0.45)	(0.50)	
Family member/friend died	0.33	0.24	0.44	-0.19**
	(0.47)	(0.43)	(0.50)	
**Anyone - Victim**				
Witnessed physical violence	0.43	0.32	0.56	-0.24***
	(0.50)	(0.47)	(0.50)	
Witnessed someone being killed	0.32	0.28	0.36	-0.08
	(0.47)	(0.45)	(0.49)	
Witnessed sexual violence	0.13	0.08	0.20	-0.12**
	(0.34)	(0.27)	(0.40)	
**Self - Perpetrator**				
Asked/forced to commit violent act	0.06	0.05	0.07	-0.02
	(0.24)	(0.23)	(0.26)	
Observations	129	74	55	129

Notes: * p<0.1, ** p<0.05, *** p<0.01. Standard deviations are in parentheses.

Table B3: Robustness Check: Main Results, Without Controls

	ACASI	Control mean (S.D.)
Panel A: Index results		
Total reported experiences	1.193	1.72
	(0.466)**	(2.38)
Reports any experiences	0.250	0.46
	(0.085)***	(0.50)
Panel B: Individual item results		
**Self - Victim**		
Seriously wounded/injured	0.082	0.03
	(0.046)*	(0.16)
Experienced physical violence	0.183	0.11
	(0.072)**	(0.31)
**Family - Victim**		
Family member/friend has been seriously wounded/injured	0.089	0.31
	(0.086)	(0.47)
Family member/friend disappeared	0.189	0.28
	(0.086)**	(0.45)
Family member/friend died	0.193	0.24
	(0.084)**	(0.43)
**Anyone - Victim**		
Witnessed physical violence	0.239	0.32
	(0.087)***	(0.47)
Witnessed someone being killed	0.080	0.28
	(0.084)	(0.45)
Witnessed sexual violence	0.119	0.08
	(0.063)*	(0.27)
**Self - Perpetrator**		
Asked/forced to commit violent act	0.019	0.05
	(0.044)	(0.23)
Controls	No	
N	129	

Notes: * p<0.1, ** p<0.05, *** p<0.01. Robust standard errors are in parentheses.

Table B4: ROBUSTNESS CHECKS

	IHS transformed	Fieldworker fixed effects		Excluding migrants	
	(1)	(2)	(3)	(4)	(5)
	Total experiences	Total experiences	Any experiences	Total experiences	Any experiences
ACASI	0.392	1.088	0.169	0.741	0.215
	(0.136)***	(0.326)***	(0.072)**	(0.290)**	(0.098)**
Controls	Yes	Yes	Yes	Yes	Yes
Control mean	0.85	1.72	0.46	0.67	0.29
Control S.D.	(1.03)	(2.38)	(0.50)	(1.34)	(0.46)
N	129	129	129	86	86

Notes: * p<0.1, ** p<0.05, *** p<0.01. Robust standard errors are in parentheses.

Table B5: Robustness Check: Placebo Conflict Items Results

	ACASI	Control mean (S.D.)	N
Panel A: Index results			
Total reported experiences	0.099	2.51	123
	(0.363)	(2.91)	
Reports any experiences	-0.028	0.59	123
	(0.066)	(0.50)	
Panel B: Individual item results			
Food shortage	-0.037	0.32	129
	(0.068)	(0.47)	
Unable to work	0.062	0.34	129
	(0.079)	(0.48)	
Unable to access health care	0.051	0.28	129
	(0.066)	(0.45)	
Unable to continue with education	-0.108	0.31	129
	(0.074)	(0.47)	
Unable to access utilities	0.052	0.36	129
	(0.047)	(0.48)	
Unable to access communications infrastructure	0.000	0.45	129
	(0.059)	(0.50)	
Asked to work for free	-0.029	0.16	129
	(0.064)	(0.37)	
Verbally threatened or insulted	-0.023	0.22	129
	(0.068)	(0.41)	
Experienced sexual violence (ACASI only)	0.041	0.04	123
	(0.052)	(0.21)	
Controls	Yes		

Notes: * $p<0.1$, ** $p<0.05$, *** $p<0.01$. Robust standard errors are in parentheses.
